# Herpes simplex virus-1 KOS-63 strain is virulent and causes titer-dependent corneal nerve damage and keratitis

**DOI:** 10.1038/s41598-021-83412-9

**Published:** 2021-02-19

**Authors:** Hamid-Reza Moein, Victor G. Sendra, Arsia Jamali, Ahmad Kheirkhah, Deshea L. Harris, Pedram Hamrah

**Affiliations:** 1 Department of Ophthalmology, Center for Translational Ocular Immunology, Tufts Medical Center, Tufts University School of Medicine, Boston, MA 02111 USA; 2 Department of Ophthalmology, Schepens Eye Research Institute/Massachusetts Eye and Ear, Harvard Medical School, Boston, MA USA; 3 Cornea Service, New England Eye Center, Tufts Medical Center, Tufts University School of Medicine, Boston, MA USA

**Keywords:** Infection, Inflammation, Herpes virus, Eye diseases

## Abstract

To investigate the acute clinical, immunological, and corneal nerve changes following corneal HSV-1 KOS-63 strain inoculation. Corneas of C57BL/6 mice were inoculated with either low dose (Ld) or high dose (Hd) HSV-1 KOS-63 or culture medium. Clinical evaluation was conducted up to 7 days post inoculation (dpi). Viral titers were assessed by standard plaque assay. Excised corneas were stained for CD45 and beta-III tubulin. Corneal flow cytometry was performed to assess changes in leukocyte subpopulations. Corneal sensation was measured using a Cochet-Bonnet esthesiometer. Naïve, sham-infected (post scarification), and McKrae-infected C57BL/6 corneas served as two negative and positive controls, respectively. Compared to Ld infected mice, Hd HSV-1 KOS-63 demonstrated higher incidence of corneal opacity (1.5 ×) and neovascularization (2.6 × ; *p* < 0.05). At 7 dpi Hd infected mice showed more severe corneal opacity (2.23 vs. 0.87; *p* = 0.0003), neovascularization (6.00 vs. 0.75; *p* < 0.0001), and blepharitis (3.11 vs. 2.06; *p* = 0.001) compared to the Ld group. At 3 dpi epitheliopathy was significantly larger in the Hd group (23.59% vs. 3.44%; *p* = 0.001). Similarly, corneal opacity was significantly higher in Hd McKrae-infected corneas as compared with Ld McKrae-infected corneas at 3 and 5 dpi. No significant corneal opacity, neovascularization, blepharitis, and epitheliopathy were observed in naïve or sham-infected mice. Higher viral titers were detected in corneas (1 and 3 dpi) and trigeminal ganglia (TG) (3 and 5 dpi) in Hd versus Ld KOS-63 groups (*p* < 0.05). Leukocyte density showed a gradual increase over time from 1 to 7 dpi in both KOS-63 and McKrae-infected corneas. Corneal flow cytometric analysis (3 dpi) demonstrated a higher percentage of Gr-1 + (71.6 vs. 26.3) and CD11b + (90.6 vs. 41.1) cells in Hd versus Ld KOS-63 groups. Corneal nerve density significantly decreased in both Hd KOS-63 and Hd McKrae infected corneas in comparison with naïve and sham-infected corneas. At 3 dpi corneal nerve density was lower in the Hd versus Ld KOS-63 groups (16.79 vs. 57.41 mm/mm2; *p* = 0.004). Corneal sensation decreased accordingly at 5 and 7 dpi in both Ld and Hd KOS-63-infected mice. Corneal inoculation with HSV-1 KOS-63 strain shows acute keratitis and nerve degeneration in a dose-dependent fashion, demonstrating virulence of this strain.

## Introduction

Herpes simplex virus type 1 (HSV-1) is a double-stranded enveloped DNA virus with a relatively large genome (152 Kb)^1,2^. HSV keratitis is the leading cause of infectious blindness in the developed world^1^, with approximately 40,000 cases of blindness or severe visual impairment occurring annually worldwide^1^. Its prevalence is estimated with 500,000 cases in the United States alone, and the annual worldwide incidence is estimated at 1.5 million^1^. During dendritic epithelial keratitis, the virus replicates in the corneal epithelium, and enters sensory nerve endings, subsequently traveling to the trigeminal ganglia (TG), where it becomes latent and can lead to recurrent keratitis upon reactivation^1–5^. Detection of HSV-1 virus in the infected epithelium by innate immune system results in the production of a variety of cytokines and chemokines, and subsequent infiltration of leukocytes and pro-angiogenic factors that result in opacification, neovascularization, and corneal scarring^5^. Animal models of corneal HSV-1 keratitis can mimic clinical and pathological aspects of human corneal HSV keratitis and have resulted in increased knowledge on the pathophysiology of this disease^6–12^.

Both human and animal studies have shown increased leukocyte infiltration^6,8,10,11,13,14^, corneal nerve degeneration, and decreased sensitivity following HSV keratitis^7,14–16^. However, animal and virus strains, as well as the viral inoculation dose have been shown to affect the severity of HSV keratitis in animal models^3,11,17,18^. To date, several HSV-1 strains have been utilized in animal studies. McKrae, RE, and KOS are among the most commonly used strains in animal models of ocular infection^6,16,17,19–23^. McKrae and RE strains have been clinically isolated from corneas of patients with HSV keratitis^21–23^, while the HSV-1 KOS-63 strain was derived in 1963 from a labial lesion of the virologist, Kendall Owen Smith^24^.

Given that the HSV-1 KOS-63 strain does not cause encephalitis and is thus not lethal following footpad inoculation, it has been considered to be avirulent^9,21,25^. The HSV-1 KOS-63 strain has been shown to express less Pro–Ala–Thr repeats in the ICP34.5 protein, important in HSV neuro-virulence, than the neurovirulent McKrae and RE HSV-1 strains, which can cause encephalitis and lead to the animal’s death^3,26^. Most of the previous studies referred to KOS-63 as KOS strain and although it has been used in ocular HSV-1 infection, controversy remains regarding the ocular virulence of this strain^9,27–32^. While some groups have shown that the HSV-1 KOS can result in stromal keratitis^9,16,27,28,33,34^, other groups have demonstrated that inoculation with the KOS strain does not result in acute or recurrent keratitis^29,30^ or stromal keratitis^31,32^. In addition, previous murine studies, in which the KOS strain was used^9,16,27,28,33,34^, did not provide detailed information on the TG infection, clinical (especially epithelial involvement) and immunological responses following HSV keratitis in the acute phase (up to 7 dpi) in C57BL/6 mice. Many transgenic mice are bred on C57BL/6 background, which has been shown to be the most resistant hosts to the HSV-1 virus^17^. Interestingly, many fluorescent recombinant HSV-1 strains are derived from the KOS parent strain. Thus, with increased use of fluorescent recombinant viral stains and transgenic mice, confirmation regarding the ocular virulence of the KOS strain in C57BL/6 mice is critical. The current study, therefore, aimed to assess the virulence of the HSV-1 KOS-63 strain in C57BL/6 mice in an acute HSV keratitis model in a titer-dependent fashion. Herein, we demonstrate that corneal inoculation of C57BL/6 mice with the HSV-1 KOS-63 strain results in corneal disease and corneal nerve damage in a titer-dependant fashion and that the virus can be detected in their TGs.

## Materials and methods

### Animals

Six-to-twleve-week-old male and female C57BL/6 mice (Charles River Laboratories, Inc, Wilmington, MA) were used in this study. All the procedures were approved by Institutional Animal Care and Use Committee of Tufts Medical Center and the Schepens Eye Research Institute. Our study was compliant with Association for Research in Vision and Ophthalmology statement for the use of animals in ophthalmic and visual research.

### Virus preparation

HSV-1 KOS-63 strain (kindly provided by Dr. Paul R. Kinchington, University of Pittsburgh, Pittsburgh, PA; GenBank accession number: JQ780693) and McKrae strain (Kindly provided by Dr. Homayon Ghiasi, Cedars-Sinai Medical Center, Los Angeles, CA; GenBank accession number: MN136524.1) were used in this study. Vero cells (African green monkey kidney cells, kindly provided by Dr. Judy Lieberman, Boston Children’s Hospital, Boston, MA) were cultured in RPMI 1640 medium with L-glutamine (Corning/Mediatech, Inc. Manassas, VA) on 6-well plates. After a monolayer was formed, the cells were infected with the virus and incubated for 48 h at 37 °C. Virus-infected Vero cells were incubated with gentleMACS dissociator (Miltenyi Biotec GmbH, Bergisch Gladbach, Germany) to extract the virus from the infected cells. High-speed centrifugation (40,000 G for 30 min at 4 °C) was applied to pellet the virus. The virus pellet was re-suspended in RPMI 1640 as above. The prepared virus was titrated using a standard plaque assay^10^ and aliquots of the virus were kept at − 80 °C for further use.

### Corneal HSV-1 inoculation and study groups

Mice were anesthetized with intraperitoneal injections of ketamine (100 mg/kg) and xylazine (10 mg/kg) mixture. A drop of 0.5% proparacaine hydrochloride (Akorn, Lake Forest, IL) was applied on the right eye to obtain local anesthesia. Right corneas were then scarified in a 5 × 5 grid pattern with a 30-gauge needle. Five microliter (µl) of RPMI containing 2 × 10^4^ (Low dose, Ld) or 2 × 10^6^ (High dose, Hd) plaque forming unit (PFU) of HSV-1 were then applied on the scarified corneas. Naïve and sham-infected mice were used as negative controls throughout the study. Sham-infected mice were scarified similar to infected mice and corneas were inoculated with 5 µl of RPMI. HSV-1 McKrae infected mice served as positive controls in this study. One dose of buprenorphine (0.1 mg/kg) was injected subcutaneously right after the corneal scarification for pain control. The studies were carried out in compliance with the ARRIVE guidelines^35^.

### Clinical grading

Mice were anesthetized at days 1, 3, 5, and 7 post-inoculation (dpi) with a ketamine/xylazine mixture and evaluated for blepharitis, corneal opacity, neovascularization, and epitheliopathy. Blepharitis was scored as 0, no puffiness of eyelids; 1, noticeably puffy lids; 2, puffy lids plus moderate crusting; 3, eyelids 50% swollen shut with severe crusting; and 4, eyelids totally shut^9^. Corneal opacity was scored using slit-lamp bio-microscopy, using a modified scale^27:^ 0, no opacity; 1, epithelial involvement of < 50%; 1.5, epithelial involvement of > 50% of the cornea; 2, stromal involvement (haze) of < 50% of the cornea and iris visible; 2.5, stromal involvement of > 50% of the cornea and iris visible; 3, stromal involvement of < 50%, iris partially invisible; 3.5, stromal involvement of > 50%, iris partially invisible; and 4, totally opaque cornea with invisible iris. Corneal neovascularization was graded from 0 to 12, based on the number of clock hours (with every 30° considered as one clock hour) that new vessels, extending from the limbus toward the cornea, occupied^36^. To assess corneal epitheliopathy we used a previously described method^37^. In brief, a 1.0 mg fluorescein sodium ophthalmic strip (FUL-GLO, Akorn, Lake Forest, IL) was diluted in 1 ml 1 × PBS (Life Technologies, Carlsbad, CA) and 5 µl of 0.1% fluorescein solution was applied on the ocular surface. After 15 s, the cornea was washed with PBS 2–3 times and using a cotton tip applicator, the excess fluid gently removed after each wash to prevent pooling of fluorescein. Photos were obtained by a Canon digital camera (PowerShot A2200), mounted on top of the slit-lamp binocular (SL-1E, Topcon, Oakland, NJ), using white light (prior to fluorescein staining) and cobalt blue light. Images were then analyzed with ImageJ software^38^ (public domain Java image processor, NIH, Bethesda, MD; https://imagej.nih.gov/ij/) to calculate the percentage of epithelial defect. For this purpose, the area of epitheliopathy, defined as the distinct fluorescein stained areas (including dendrites), was selected using polygon selection tool and measured first. Then the epitheliopathy area was divided by the area of the whole cornea, measured by selection of the whole cornea using oval selection tool. Clinical grading was performed by 3 masked observers.

### Viral titration of corneas and trigeminal ganglia

We pooled and homogenized 2 corneas or 2 ipsilateral TGs from 2 infected mice in 300 µl of RPMI using gentle MACS dissociator for each experiment. A standard plaque assay^10^ was performed to titer the virus. In brief, serial dilutions of the homogenized supernatants were added in duplicate to the monolayer of Vero cells in 6 well plates. Plates were incubated at 37 °C for 1 h. Subsequently, solution of 0.5% methylcellulose (Sigma-Aldrich, St. Louis, MO) and 5% fetal bovine serum (FBS; Gemini BioProducts, West Sacramento, CA) in Dulbecco’s Modified Eagle’s Medium (DMEM, Corning/Mediatech, Inc., Manassas, VA) were added to each well and plates were incubated again at 37 °C. After 48–72 h, well contents were vacuumed and 1 ml of 1% crystal violet (Sigma-Aldrich) was added to each well. After 30 min plates were washed with PBS and number of white spots was counted to calculate the virus titer as PFU/ml (detection limit of 100 PFU/ml).

### Corneal whole-mount immunofluorescent staining

Harvested corneas were fixed in chilled acetone for 15 min at − 20 °C and washed 3 times in PBS for 10 min each. Corneas were blocked in 3% bovine serum albumin containing 1% anti-FcR monoclonal antibody (CD16/CD32; BioXCell, West Lebanon, NH) for 60 min at room temperature. Corneas were then stained for PE-conjugated rat anti-mouse CD45 (30-F11) (Biolegend, San Diego, CA) and NL637-conjungated mouse anti-βIII-Tubulin (R&D, Minneapolis, MN, USA) overnight at 4 °C on the shaker. Corneas were washed 3 times in PBS for 10 min each, flattened after 3 radial incisions, and mounted on a slide using 4′, 6-diamidino-2-phenylindole (DAPI) mounting media (Vectashield, Vector Laboratories, Inc. Burlingame, CA, USA).

### Confocal microscopy and image analysis

The whole-mounted corneas were imaged using a confocal Leica TCS SP5 (Leica Microsystems, Wetzlar, Germany). Central and peripheral areas of each cornea were assessed separately, with the central area defined as the area within 0.6 mm of the corneal center, and the periphery defined as being within a 0.9–1.5-mm radial distance from the center. Three different non-overlapping fields from the peripheral cornea and one central field were imaged for each cornea. At least three different corneas were stained and imaged per group. Stacked images were analysed off-line with IMARIS software version 8.0 (Bitplane AG, Zurich, Switzerland) to calculate the number of CD45 (pan-leukocyte marker) positive cells. The number of cells in peripheral corneal fields was then averaged. Corneal nerves were traced using NeuronJ^39,40^ (http://www.imagescience.org/meijering/software/neuronj/), a free semi-automatic software. Nerve density was reported as mm/mm^2^.

### Flow cytometry

At 3 dpi, infected corneas were excised and digested in DNAse I (2 mg/ml) and collagenase D (2 mg/ml) (Roche Diagnostics GmbH, Manheim, Germany) in RPMI for 30 min at 37 °C. Single-cell suspensions were filtered through a 70 µm cell strainer (VWR International, LLC, Radnor, PA) and washed with PBS to stop the enzyme reaction. The remaining tissues were further digested at 37 °C for additional 30 min. Remaining single cells were filtered and washed again and added to the previously digested cells. Similarly, both corneas of wild-type C57BL/6 mice were excised and single cells were obtained. Corneal single cell suspensions were blocked in 1% anti-FcR monoclonal antibody in PBS for 15 min at 4 °C to minimize non-specific staining and then further incubated with the desired conjugated antibodies for 1 h at 4 °C. Conjugated antibodies used for immunohistochemistry staining included: Pacific Blue rat anti-mouse CD45 (30-F11), phycoerythrin (PE)-conjugated mouse anti-mouse NK-1.1 (PK136), PE-conjugated rat anti-mouse Gr-1 (RB6-8C5), Alexa fluor-647-conjugated rat anti-mouse F4/80 (BM8), and Alexa fluor-647-conjugated rat anti-mouse/human CD11b (M1/70) all from Biolegend. Fluorescein isothiocyanate (FITC)-conjugated rat anti-mouse CD11c (HL3) was purchased from BD Pharmingen (San Diego, CA). Isotype controls included Pacific Blue rat IgG2b, κ (RTK4530), Alexa fluor-647-conjugated rat IgG2a, κ (RTK2758), PE-conjugated rat IgG2b, κ (RTK4530), FITC Armenian Hamster IgG (HTK888), and Alexa fluor-647-conjugated rat IgG2b, κ (RTK4530) all from Biolegend. Stained corneal single cell suspensions were washed with PBS, centrifuged (1300 RPM for 10 min) and re-suspended in 4% paraformaldehyde. The stained cells were analyzed using a BD LSR II flow cytometer (BD Biosciences, San Jose, CA). FlowJo software (FlowJo, LLC, Ashland, OR) was used for additional cell analysis. Doublet cells were excluded by eliminating disproportions between forward scatter-width (FSC-W) versus forward scatter-height (FSC-H). Gates were plotted based on appropriate isotype control staining for each antibody.

### Corneal sensation

Corneal nerve sensitivity was measured by a Cochet-Bonnet esthesiometer (Luneau Technology, Pont-de-l’Arche, France). In brief, the mono-filament was applied to the central cornea of un-anesthetized mice, avoiding contact with whiskers and eyelashes. Maximum mono-filament length is 6 cm; the micro-filament length required to measure a blink reflex was recorded. The procedure was repeated three times to ensure reproducibility and average length was used for analysis.

### Statistical analysis

Statistical analysis was performed with Prism version 6.01 (GraphPad Software, Inc., La Jolla, CA). We used chi square test to compare incidence percentage of corneal opacity, blepharitis, and neovascularisation between two different groups of infected mice. Student’s t-test was used to compare means between the two groups and one-way ANOVA, followed by Tukey’s test used to compare the means among three or more groups. Results were shown as mean ± standard error of the mean (SEM) and *p* values of < 0.05 were considered as statistically significant.

## Results

### Titer-dependent incidence and clinical severity in mice infected with Ld (2 × 10^4^ PFU) versus Hd (2 × 10^6^ PFU) HSV-1 KOS-63

Ten to 17 mice at each time point were evaluated for clinical signs of the disease in either 2 × 10^4^ PFU (Ld) or 2 × 10^6^ PFU (Hd) KOS-63-infected mice. At the end of the follow-up period (7 dpi), the incidence of blepharitis did not show a significant difference between the two doses of the virus (93.7% and 94.4% in Ld and Hd KOS-63-infected mice, respectively, *p* = 0.93). However, the incidence of mice, which developed corneal opacity was significantly higher in the Hd KOS-63-infected mice (88.2%) as compared to the Ld KOS-63-infected mice (56.2%), *p* = 0.03. The incidence of corneal neovascularisation was also significantly higher in the Hd KOS-63-infected mice (81.8%), as compared to the Ld KOS-63-infected mice (31.2%), *p* = 0.009.

**Figure 1:**
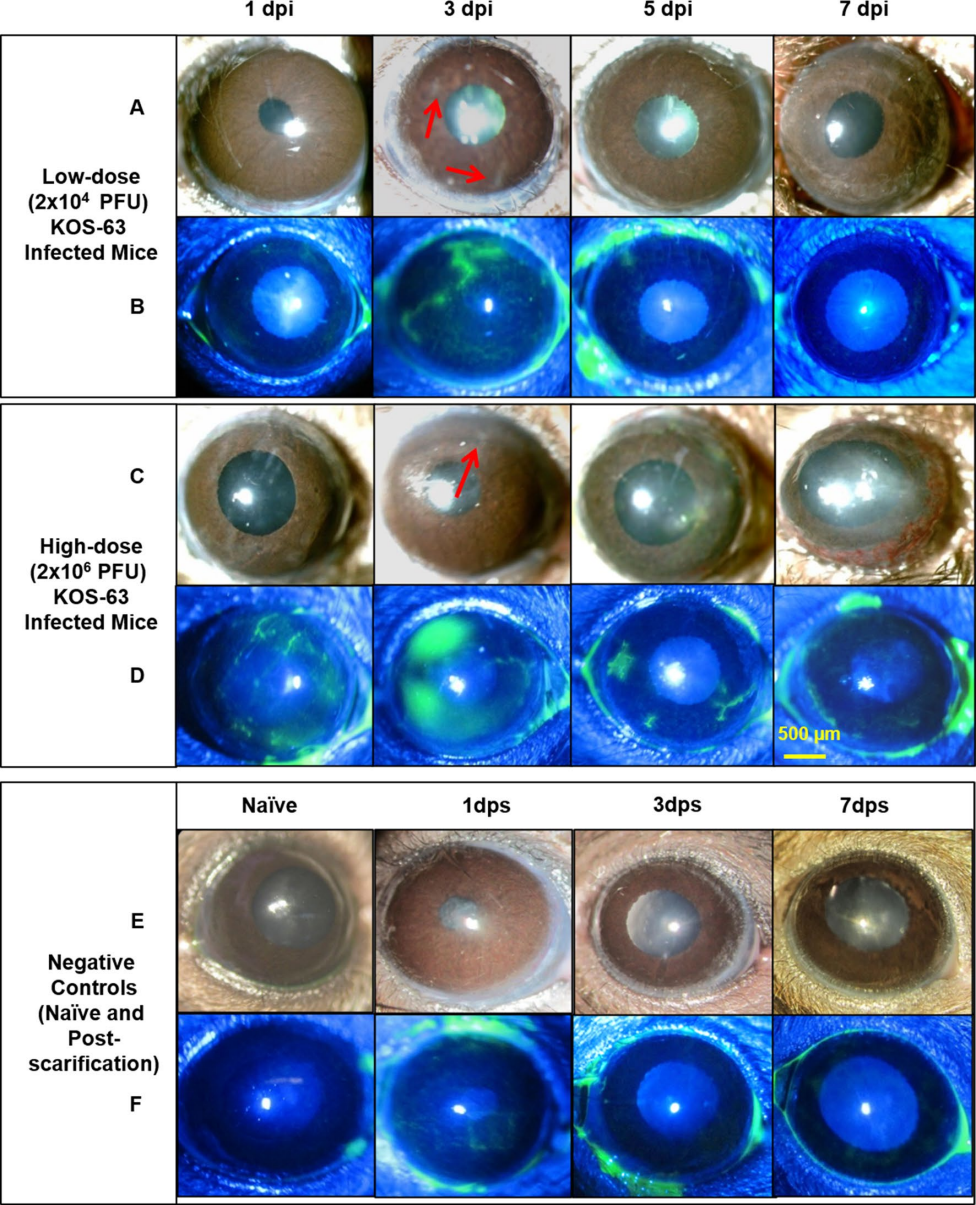
Representative slit-lamp photos following corneal inoculation with low dose (Ld, 2 × 10^4^ PFU; **A, B**), high dose (Hd, 2 × 10^6^ PFU; **C, D**) HSV-1 KOS-63 strain, and negative controls (naïve and sham-infected; E, F). Representative white light images from Ld (**A**) and Hd (**C**) inoculated corneas at 1, 3, 5, and 7 days post infection (dpi) demonstrate the corneal opacity at these time points. Hd HSV-1 infected mice developed more severe corneal opacity as compared to Ld infected mice. Red arrows are showing the opacity at 3 dpi. Representative cobalt blue filtered light images from Ld (**B**) and Hd (**D**) inoculated corneas demonstrate corneal epithelial defect following staining the corneas with 0.1% fluorescein at 1, 3, 5, and 7 dpi. In both Ld and Hd KOS-63 infected mice corneal epithelial defects peaked at 3 dpi. Representative white light (**E**) and blue filtered (**F**) corneal images in naïve and 1,3, and 7 days post scarification (dps).

**Figure 2:**
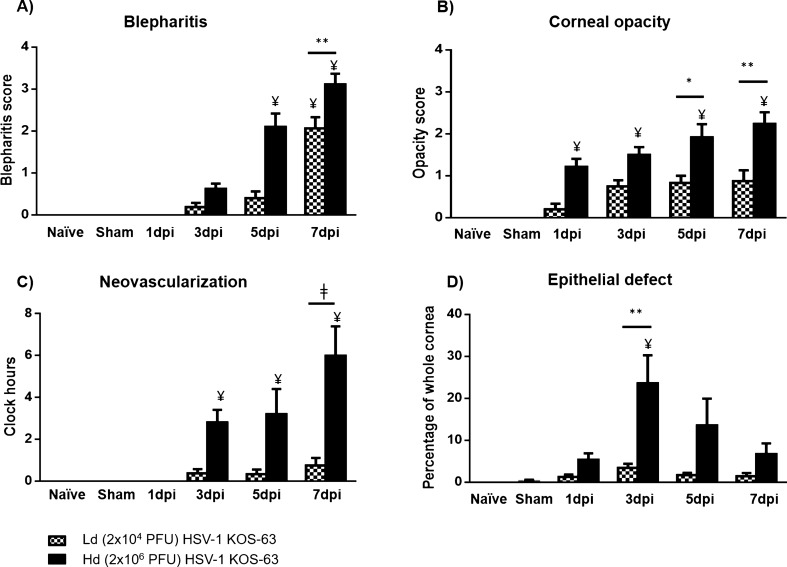
Comparison of clinical scores in naïve, sham-infected (1-day post scarification (dps)), and following corneal infection with Ld and Hd HSV-1 KOS-63 strain at 1, 3, 5, and 7 days post infection (dpi). (**A**) Blepharitis scores. At 1 dpi no blepharitis was observed but it increased gradually in both Hd and Ld infected mice. Blepharitis score was significantly higher in the Hd infected mice compared to Ld infected mice at 7 dpi (**p = 0.001). No blepharitis was observed in naïve and sham infected mice (1–7 dps), 1 dps is presented as representative. (**B**) Corneal opacity scores are demonstrated in both Ld and Hd infected mice. Opacity scores were higher in the Hd infected mice as compared to Ld infected mice and was significant at 5 dpi (*p = 0.04) and 7 dpi (**p = 0.003). No corneal opacity was observed in naïve and sham infected mice (1–7 dps), 1 dps is presented as representative. (**C**) Corneal neovascularization scores according to the clock hours area of vessel coverage. Neovascularization was more significant in the Hd infected mice compared to Ld infected mice at 7 dpi (ǂ*p* < 0.0001). No corneal neovascularization was observed in naïve and sham-infected mice (1–7 dps), 1 dps is presented as representative. (**D**) Percentage of corneal epithelial defect was higher in the Hd infected mice compared to Ld infected mice in all time points and was significant at 3 dpi (**p = 0.001). No corneal opacity was observed in naïve mice. Minor epithelial defect remained after corneal scarification only at 1 dps, which was not statistically significant compared to naïve mice. Bars are showing mean ± SEM. ANOVA: *p* < 0.0001. ¥, demonstrates the statistical significance (*p* < 0.05) between the marked column and naïve and sham-infected. *p* values are calculated by one-way ANOVA followed by Tukey’s multiple comparison test.

Representative white light and cobalt blue filtered images for comparison of corneal opacity and epitheliopathy in the Ld and Hd HSV-1 KOS-63 infected corneas, as well as naïve and sham-infected mice at different time points are illustrated in Fig. 1. Blepharitis was not present at 1 dpi in any of the groups; however, at 3 dpi blepharitis scores were 0.18 ± 0.10 and 0.62 ± 0.12 in the Ld- and Hd-infected mice, respectively (*p* = 0.68). At 7 dpi, blepharitis scores increased significantly to 2.06 ± 0.26 and 3.11 ± 0.25 in the Ld- and Hd-infected mice, respectively (*p* = 0.001). Blepharitis scores showed an increasing trend from 1 to 7 dpi (ANOVA: *p* < 0.0001) (Fig. 2A).

Corneal opacity scores continuously increased in both groups from 1 to 7 dpi (ANOVA: *p* < 0.0001). Corneal opacity was significantly higher in the Hd KOS-63-infected mice as compared to Ld KOS-63-infected mice at 7 dpi (2.23 ± 0.27 vs. 0.87 ± 0.25, respectively; *p* = 0.0003) (Fig. 2B). Further, corneal neovascularization increased significantly from 1 to 7 dpi in both groups, and was more prominent in the Hd compared to the Ld KOS-63-infected at all time points (ANOVA: *p* < 0.0001). Similar to HSV-1 KOS-63-infected mice, corneal opacity was higher in Hd as compared to Ld HSV-1 McKrae-infected mice (Supplementary Fig. 1). There was no significant difference among Hd KOS-63 vs. Hd McKrae infected corneas at 1, 3, and 5 dpi (*p* > 0.99). At 5 dpi, corneal opacity was significantly higher in Hd McKrae corneas as compared to Ld KOS-63 corneas (0.83 ± 0.16 vs. 2.0 ± 0.0; *p* = 0.029) (Supplementary Fig. 1).

At 7 dpi, the time point with highest neovascularization, 6.00 ± 1.39 and 0.75 ± 0.35 clock hours of the Hd and Ld KOS-63-infected corneas demonstrated presence of vessels, respectively (*p* < 0.0001) (Fig. 2C). Moreover, epithelial defects were larger in the Hd as compared to the Ld KOS-63-infected mice at all time points, but were only significant at 3 dpi (23.59 ± 6.67% vs. 3.44 ± 0.96%, respectively; *p* = 0.001) (Fig. 2D). No opacity, neovascularization, and blepharitis were observed in naïve and sham-infected mice (1, 3, and 5 days post-scarification; Fig. 2 and Supplementary Fig. 2). Blepharitis scores were significantly higher starting at 5 dpi in the Hd KOS-63-infected mice, as compared to naïve and sham-infected mice (Fig. 2A). Opacity scores were significantly higher only in the Hd KOS-63-infected mice, than in naïve and sham-infected mice (Fig. 2B). Neovascularization was significantly increased only in Hd KOS-63 infected mice between 3 and 7 dpi, as compared to controls (Fig. 2C). Very minor epithelial changes were present in sham-infected corneas (only at 1-day post-scarification) (0.34 ± 0.27% vs. 0.0% in naïve mice; *p* = 0.99) (Fig. 2D and Supplementary Fig. 2).

### HSV-1 KOS-63 strain is capable of infecting corneas and ipsilateral trigeminal ganglia

**Figure 3:**
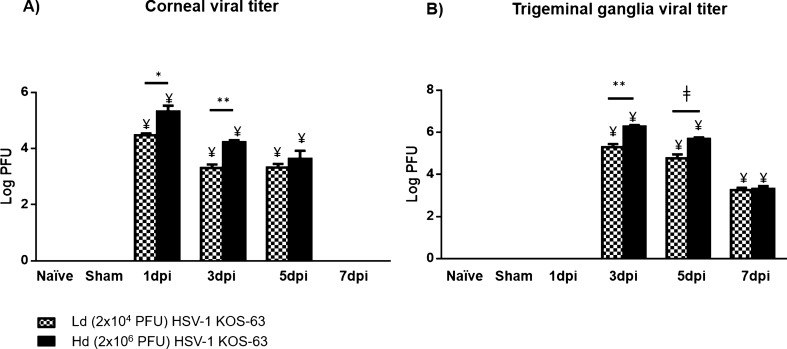
The viral titer levels in corneas and trigeminal ganglia (TG) of Ld and Hd HSV-1 KOS-63 infected mice at 1, 3, 5, and 7 days dpi. Corneal and TG of naïve and sham-infected mice served as controls. (**A**) Mean viral titer in corneas; Hd infected mice had higher viral titer at 1dpi (*p = 0.004) and 3 dpi (**p = 0.001). (**B**) Mean viral titer in the TGs; It demonstrated higher titers in the Hd-infected mice compared to Ld-infected mice at 3 dpi (**p = 0.0008) and 5 dpi (ǂ*p* < 0.0001). At each time point 6–10 TGs or corneas, from 6 to 10 mice and from 3 to 4 independent experiments, were homogenized and tittered in pairs (2 corneas or 2 TGs were homogenized in a same tube for titration). Bars are showing mean ± SEM. ANOVA: *p* < 0.0001. ¥, demonstrates the statistical significance (*p* < 0.05) between the marked column and naïve and sham-infected. *p* values are calculated by one-way ANOVA followed by Tukey’s multiple comparison test.

In infected corneas, the maximum viral load was detected at 1 dpi but it was undetectable at 7 dpi in both Hd and Ld KOS-63-infected groups. However, the corneal viral titer was significantly higher in the Hd compared with the Ld KOS-63-infected mice at 1 dpi (5.32 ± 0.20 vs. 4.47 ± 0.06 Log PFU, respectively; *p* = 0.004) and 3 dpi (4.22 ± 0.07 vs. 3.30 ± 0.12 Log PFU, respectively; *p* = 0.001) (Fig. 3A). Interestingly, corneal viral titers in Hd McKrae-infected mice were 4.82 ± 0.02 Log PFU at 7 dpi, as compared to undetected levels in Ld and Hd KOS-63-infected mice (*p* < 0.0001; Supplementary Fig. 2A). While at 1 dpi, viral titers in TG of infected mice were not detectable, viral titers in TGs significantly increased at 3 and 5 dpi in the Hd KOS-63-infected group (6.27 ± 0.06 and 5.68 ± 0.07 Log PFU, respectively) compared with Ld KOS-63-infected group (5.29 ± 0.14 and 4.76 ± 0.18 Log PFU) (both *p* < 0.001). However, at 7 dpi viral titers reached similar levels in the Hd and Ld KOS-63-infected mice (3.37 ± 1.58 and 3.30 ± 1.51 Log PFU, respectively) (Fig. 3B). TG viral titers in Ld and Hd KOS-63-infected mice were significantly higher than in McKrae-infected mice at 3 dpi (3.07 ± 0.08 Log PFU; *p* < 0.0001 for both), but similar at 7 dpi (Supplementary Fig. 2B). Naïve and sham-infected controls were negative (Fig. 3).

### Immune cell infiltration in acute herpes simplex keratitis induced by HSV-1 KOS-63 strain

**Figure 4:**
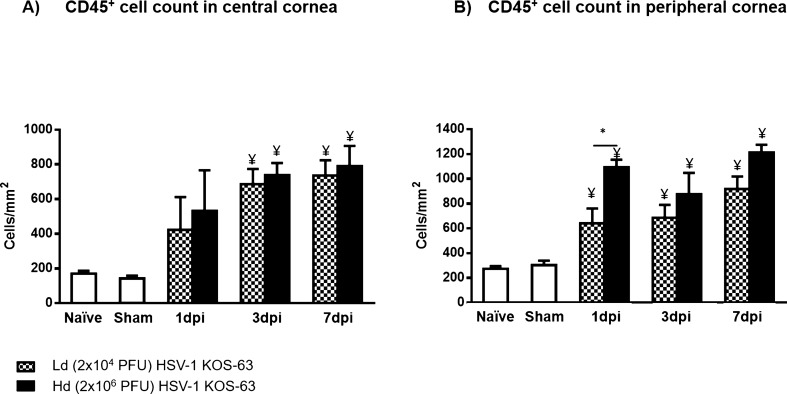
Number of CD45 + inflammatory cells in the central (**A**) and peripheral (**B**) cornea in naïve, sham-infected, Ld and Hd KOS-63-infected mice. Whole mount confocal images of corneas, which were stained with PE-conjugated rat anti-mouse CD45 (30-F11) antibody, were analyzed with IMARIS software to calculate the number of CD45 + cells in the cornea. At each post infection timepoint 3–9 corneas were stained. In addition, 6 and 9 naïve and sham-infected (1 day post scarification) corneas were stained, respectively. Bars are showing mean ± SEM. ANOVA: *p* < 0.0001; *p = 0.01, calculated by one-way ANOVA followed by Tukey’s multiple comparison test. ¥, demonstrates the statistical significance (*p* < 0.05) between the marked column and the sham-infected controls.

The number of bone marrow (BM)-derived immune cells (CD45^+^ cells), identified by immunohistochemistry staining in the central and peripheral corneas of naïve, sham control, and HSV-1 KOS-63-infected mice at 1, 3, and 7 dpi have been demonstrated in Fig. 4. At 3 and 7 dpi, CD45^+^ cell density increased in the central cornea of both Ld (686.5 ± 8.50 and 735.9 ± 88.89 cells/mm^2^, respectively) and Hd (738.8 ± 68.85 and 790.0 ± 116.8 cells/mm^2^, respectively) KOS-63-infected mice compared to naïve (170.7 ± 16.04 cells/mm^2^) and sham-infected (143.1 ± 16.15 cells/mm^2^) mice (all *p* < 0.05). However, there was no significant difference in the CD45^+^ cell density in the central cornea between Ld and Hd KOS-63-infected groups at any time point (all *p* > 0.05; Fig. 4A). In both Ld and Hd KOS-63-infected groups, the number of CD45^+^ cells in the peripheral cornea increased significantly at 1 dpi (639.4 ± 121 and 1092 ± 61.66 cells/mm^2^, respectively), as compared to naïve (273.2 ± 21.92 cells/mm^2^; *p* = 0.03 and *p* < 0.0001, respectively) and sham-infected mice (334.4 ± 34.16 cells/mm^2^; *p* = 0.02 and *p* < 0.0001, respectively), and remained elevated until 7 dpi. The density of CD45^+^ cells significantly increased in the Hd-infected group compared to the Ld-infected group at 1 dpi (*p* = 0.01) (Fig. 4B). Additionally, we compared CD45^+^ cell density in central and peripheral cornea between HSV-1 KOS-63 and HSV-1 McKrae-infected mice. CD45^+^ cell density was significantly increased in the central corneas of McKrae-infected mice (2088 ± 180.4 cells/mm^2^) at 7 dpi, as compared to Ld and Hd KOS-63-infected mice (*p* < 0.0001 for both; Supplementary Fig. 3A). CD45^+^ cell density in the corneal periphery was significantly lower at 1 dpi (291.0 ± 32.85 cells/mm^2^) and 3 dpi (361.8 ± 37.71 cells/mm^2^) in McKrae-infected corneas as compared to similar timepoints in Hd KOS-63-infected corneas (*p* < 0.001 and *p* = 0.045, respectively; Supplementary Fig. 3B). However, at 7 dpi CD45^+^ cell density in peripheral corneas was significantly higher in McKrae-infected mice (3925.0 ± 230.1 cells/mm^2^), as compared to both Ld and Hd KOS-63-infected mice (*p* < 0.0001 for both; Supplementary Fig. 3B).

**Figure 5:**
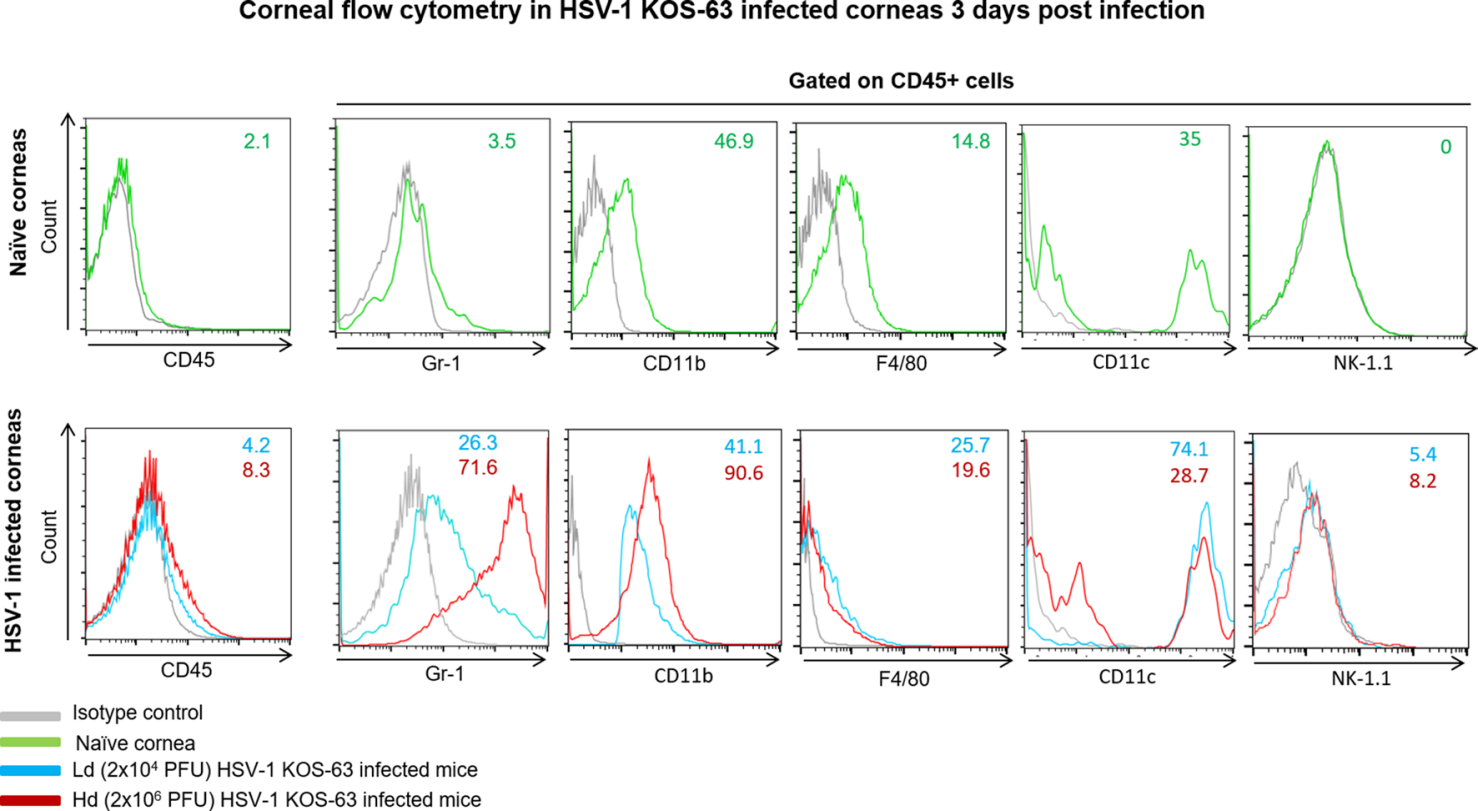
Flow cytometry histograms of Ld and Hd HSV-1 KOS-63-infected corneas at 3 dpi (lower row) compared with naïve corneas (upper row). Histograms are representative from 2 independent experiments. In one experiment corneas of 6 Hd- and 6 Ld-infected C57BL/6 mice were pooled and digested separately. In the other experiment 27 corneas of naïve C57BL/6 mice harvested and digested together in a same fashion. Single cell suspensions were stained for different cell markers. Gating was performed using isotype control antibodies. CD45^+^ population was selected in each group initially and percentage of CD45^+^ cells to the total number of cells is demonstrated in the histograms. Percentage of Gr-1^+^ cells, CD11b^+^ cells, and F4/80^+^ were demonstrated after gating on CD45^+^ cell population. In the lower row blue line demonstrates the Ld and red line demonstrates the Hd HSV-1 KOS-63-infected mice. Gray line represents isotype control in all histograms.

To further analyze the subpopulation of infiltrated cells in corneas of HSV-1 KOS-63 strain infected mice, we used flow cytometry. The percentage of CD45 + cells in the cornea increased to 4.2% and 8.3% in Ld and Hd groups, respectively, compared to naïve corneas (2.1%). We then gated on CD45^+^ cells and compared the percentage of other immune cell subpopulations between naïve and infected mice (Fig. 5). While Gr-1^+^ cells constituted 3.5% of leukocytes (CD45^+^ cells) in naïve corneas, their percentage increased to 26.3% and 71.6% in the Ld and Hd infected mice, respectively. 41.1% and 90.6% of CD45^+^ cells expressed CD11b^+^ in the Ld and Hd infected groups, respectively. In addition, F4/80^+^ cells constituted 25.7%, and 19.6% of CD45 + cells in Ld- and Hd-infected mice, respectively (Fig. 5). Furthermore, 74.1% and 28.7% of corneal CD45^+^ cells expressed CD11c^+^ in Ld- and Hd-infected mice, respectively. Moreover, natural killer cells (CD45^+^ / NK-1.1^+^) were not detected in naïve corneas, while in the Ld and Hd infected corneas these cells constituted 5.4% and 8.2% of the CD45^+^ cells, respectively (Figure not shown). In summary, at 3 dpi the percentage of CD45^+^ cells was 2 times higher in the Hd-infected corneas as compared to Ld-infected. Moreover, gating on CD45^+^ cells, Gr-1^+^, CD11b^+^, and NK cell percentage were higher in the Hd- versus Ld-infected corneas. Interestingly, CD11c^+^ cells constituted majority of CD45^+^ cells in the Ld-infected corneas at 3 dpi and had higher percentage compared to Hd-infected (Fig. 5). Different immune cell subpopulation at 3 dpi in McKrae-infected corneas are demonstrated in Supplementary Fig. 3C. The majority of corneal immune cells in McKrae-infected corneas were Gr-1^+^ cells (48.2%).

### HSV-1 KOS-63 strain results in nerve damage after corneal inoculation

**Figure 6:**
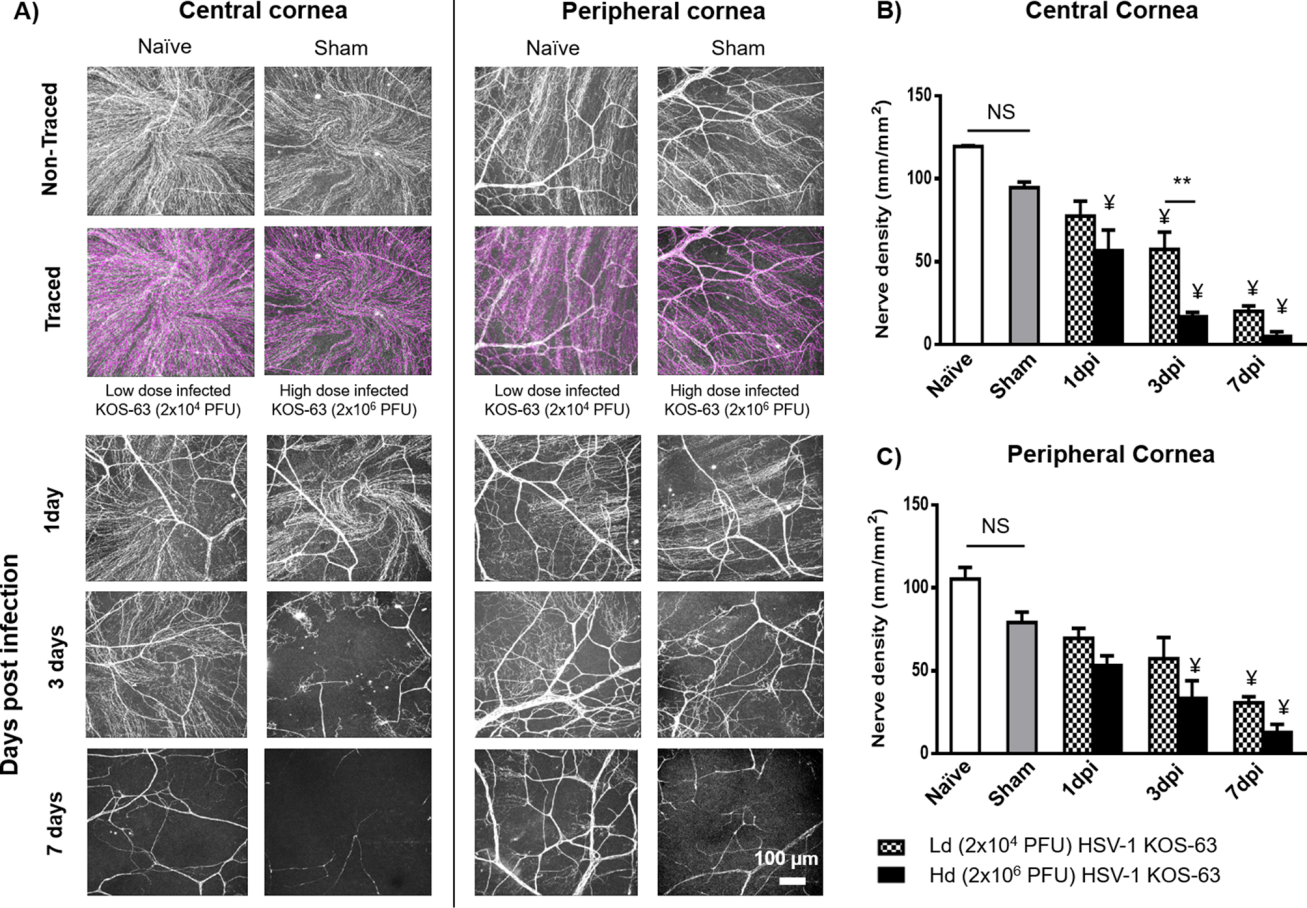
Corneal nerve density in the center and periphery of naïve and sham-infected corneas as well as Ld and Hd HSV-1 KOS-63-infected corneas. (**A**) Sample confocal micrographs of whole mount corneas stained with NL637 conjugated beta III tubulin antibody. There is a non-significant corneal nerve loss in the sham control group compared with naïve corneas. Sample central and peripheral corneal nerve tracing in naïve and sham-infected mice is demonstrated using NeuronJ software. Diminished corneal nerves in the infected corneas were observed at 1, 3, and 7 dpi especially in the central cornea of Hd infected mice. (**B**) Corneal nerve density (mm/mm^2^) was calculated with NeuronJ software after tracing the nerves in whole mount confocal micrographs. (**C**) Corneal nerve density (mm/mm^2^) in the peripheral cornea was calculated the same as central cornea. Each group is demonstrating the average of at least 3 whole mount stained corneas. Naïve and sham-infected central and peripheral corneal nerves were not significantly different although there was a trend towards more corneal nerve loss in the sham-infected group compared to the naïve mice corneas. ANOVA: *p* < 0.0001; *p = 0.01; **p = 0.004 was calculated by one-way ANOVA followed by Tukey’s multiple comparison test. ¥, demonstrates the statistical significance (*p* < 0.05) between the marked column and the sham-infected controls.

Figure 6A demonstrates representative confocal microscope micrographs of βIII-tubulin stained central and peripheral corneal nerves in naïve, sham-infected, and Hd or Ld HSV-1 KOS-63-infected mice. Total nerve density did not significantly decrease in sham-infected mice compared to naïve mice in either the central (94.72 ± 4.07 vs. 119.46 ± 0.58 mm/mm^2^; *p* = 0.23) or peripheral corneas (79.12 ± 7.70 vs. 105.28 ± 8.61 mm/mm^2^; *p* = 0.39). At 1 dpi, there was no significant loss of nerves in the central cornea of Ld KOS-63-infected mice, as compred to sham-infetced mice. However, the central corneal nerve density significantly decreased at 3 and 7 dpi for the Ld and for all timepoints for the Hd KOS-63-infected mice, as compared to sham-infected (*p* < 0.05) (Fig. 6B). As early as 3 dpi, the central corneal nerve density was significantly lower in the Hd compared with Ld KOS-63-infected mice (16.79 ± 3.17 vs. 57.41 ± 14.64 mm/mm^2^, respectively; *p* = 0.004) (Fig. 6B). Peripheral corneal nerves further decreased significantly at 7 dpi in the Ld KOS-63-infected mice (30.87 ± 3.61 mm/mm^2^) compared to sham-infected (*p* = 0.005). Morevoer, in the Hd KOS-63-infected corneas, peripheral corneal nerves decreased significantly at 3 dpi (33.48 ± 12.99 mm/mm^2^) and 7 dpi (12.79 ± 8.55 mm/mm^2^) compared to sham-infected (*p* = 0.01 and *p* < 0.0001, respectively) (Fig. 6C). Interestingly, there was no significant difference in peripheral corneal nerve density between the Ld and Hd KOS-63-infected groups (Fig. 6C). However, we did observe a linear decrease in the central and peripheral corneal nerve densities during the 7 days follow-up period in both Ld and Hd KOS-63-infected mice (ANOVA: *p* < 0.0001). Similar to Hd KOS-63-infected corneas, central corneal nerve density was significantly reduced at 1 dpi in Hd McKrae-infected corneas (62.20 ± 4.32 mm/mm^2^), as compared to naïve and sham-infected controls. (*p* = 0.0003 and *p* = 0.02, respectively (Supplementary Fig. 4).

**Figure 7:**
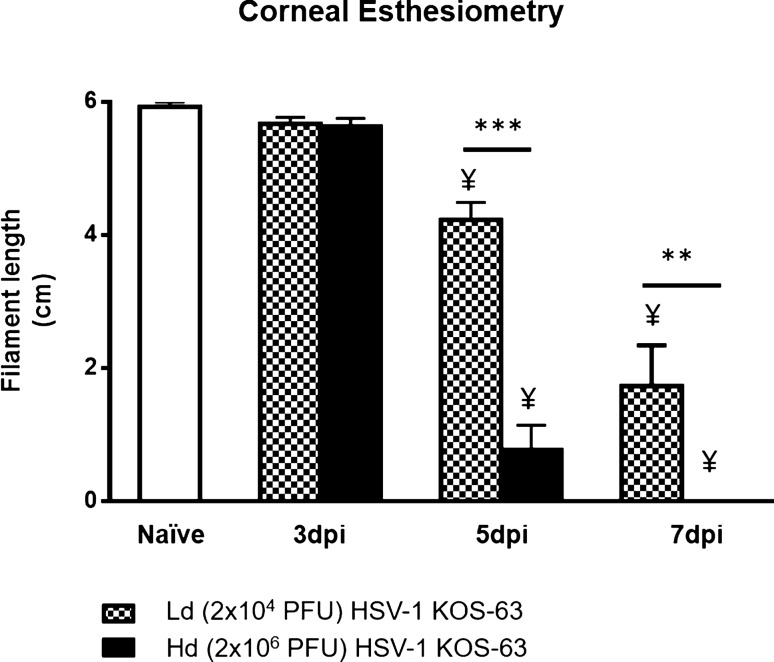
Corneal esthesiometry results. Corneal sensitivity was measured using Cochet-Bonnet esthesiometer in the central corneas. Maximum filament length is 6 cm and indicates full sensitivity. Corneal nerve sensitivity was significantly decreased after 5 dpi in both low dose (Ld) and high dose (Hd) HSV-1 KOS-63-infected corneas as compared to naïve corneas. Bars are showing mean ± SEM. *p* values are calculated by one-way ANOVA followed by Tukey’s multiple comparison test. ***p < 0.0001, **p = 0.007. ¥, demonstrates the statistical significance (*p* < 0.05) between the marked column and naïve cornea.

Additionally, corneal esthesiometry confirmed significantly decreased corneal nerve sensitivity, starting from 5 dpi in both Ld and Hd HSV-1 KOS-63-infected corneas. At 5 dpi corneal nerve sensitivity was significantly lower in the Hd-infected corneas, as compared to Ld-infected corneas (filament length of 0.77 ± 0.37 cm vs. 4.23 ± 0.26 cm; *p* < 0.0001). Similarly, at 7 dpi, corneal nerve sensitivity was significatly lower in the Hd-infected corneas as compared to Ld-infected corneas (filament length of 0.0 ± 0.0 cm vs. 1.73 ± 0.61 cm; *p* = 0.007) (Fig. 7).

## Discussion

Herein, we demonstrate that the HSV-1 KOS-63 strain can cause a titer-dependant keratitis in C57BL/6 mice when applied topically after corneal scarification. Although the HSV-1 KOS cannot infect the central nervous system and cause encephalitis when applied peripherally, through corneal inoculation^9,21,25^, we show that it can infect the corneal nerves as part of the peripheral nervous system, and subsequently migrate to the TGs. This is further demonstrated by corneal nerve degeneration observed in our studies. While development of stromal keratitis following inoculation with KOS strain in C57BL/6 mice has been recently reported, the presence of the virus in the TGs or corneal leukocyte and nerve alterations have not been previously assessed^41^. However, in contrast, Nguyen et al.^32^ did not observe stromal keratitis following ocular inoculation of HSV-1 KOS on scarified C57BL/6 mice.

We show that Hd KOS-63 strain not only increases the frequency of corneal opacity and neovascularization, but also results in increased severity of ocular manifestations as compared to Ld of virus. Similarly, using McKrae strain as a positive control we observed a titer-dependent keratitis. Consistent with our study, higher doses of HSV-1 strain RE demonstrated more severe corneal neovascularization in BALB/c mice at 15 dpi^42^. Moreover, Zheng et al.^43^ demonstrated that 2.5 × 10^7^ PFU of HSV-1 McKrae caused significantly higher corneal opacity scores than a 100 times lower dose. However, albeit higher dose of HSV-1 KOS-63 (5 × 10^4^ PFU vs. 2.5 × 10^4^ PFU) increased the incidence of herpetic stromal keratitis in BALB/c mice, it did not cause more severe keratitis^27^. Corneal infection using other HSV-1 strains on mice (CJ394, OD4, 994, 401, 394)^9,18^ or rabbits (RE and McKrae)^22^ did not demonstrate any titer-dependant severity of the disease. Corneal epithelial defects at 3 dpi were at its highest for both Hd and Ld HSV-1 KOS-63-infected corneas; this is in accordance with a significant rise of inflammatory cells (CD45^+^ cells) in the central cornea at 3 dpi, as compared to naïve corneas. Significantly larger epithelial defects were observed in the Hd HSV-1 KOS-63 infected corneas as compared to the Ld group. Higher corneal viral titer in the Hd KOS-63 infected group may, in part, explain the increased severity of clinical signs observed in this group, as compared to Ld KOS-63 infected group. Similarly, mice that developed herpetic stromal keratitis (HSK), using RE strain, showed higher viral titer in their tear films (although not significant) compared to those without HSK^44^.

We demonstrate increased number of infiltrating leukocyte (CD45^+^ cells) in corneas of both KOS-63 infected groups. We have previously shown an increased influx of CD45^+^ cells in the cornea after HSV-1 McKrae infection^10,45^. Here we show that although KOS-63-infected corneas have higher number of CD45^+^ cells at 3 and 5 dpi compared to McKrae infected mice, their density is lower at 7 dpi, suggesting an earlier, but less severe inflammatory response with KOS-63 infection. Interestingly, analysis of corneal leukocyte subpopulations is different between Hd and Ld KOS-63-infected mice. CD11b^+^ cells are suggested to be involved in causing blepharitis in HSV-1 infected mice^8^. Therefore, a higher percentage of CD11b^+^ cells in the Hd KOS-63-infected mice might explain the more severe blepharitis in the Hd as compared to the Ld KOS-63-infected group. Neutrophils are a source of vascular endothelial growth factor (VEGF)-A and have a crucial role in inducing angiogenesis following HSV-1 ocular infections^46^. Interestingly, we show a larger infiltration of CD45^+^ Gr-1^+^ cells into the cornea of the Hd KOS-63-infected mice (71.6%) as compared to the Ld-infected group (26.3%), which can explain the higher neovascularization score in the former group. NK cells and macrophages play a role in the control and clearance of the virus from the cornea^6^. Percentages of both cells increase at 3 dpi as compared to naïve mice. CD11c^+^ DCs are believed to control the virus replication in the cornea indirectly by increasing migration of NK cells to the center of cornea^47^ and by systemic dissemination of the virus to the TGs, thus decreasing the virus titer in the cornea^10^. Interestingly, we have recently demonstrated that depletion of CD11c^+^ cells resulted in more severe acute HSV keratitis^10^. The current study shows that Hd-infected mice have a lower frequency of CD11c^+^ cells among CD45^+^ immune cells (28.7%) as compared to Ld-infected mice (74.1%) at 3 dpi, which can partly explain the higher viral titer in the cornea and more sever keratitis in the Hd-infected mice. It is plausible that in the presence of a lower viral load, DCs can increase and protect the cornea from severe damage, while a higher viral load could prevent increased DC density and therefore cause more severe clinical symptoms.

Our data shows that both central and peripheral subbasal corneal nerve density decreases gradually over the first 7 days after inoculation, with more severe loss in the central cornea and in Hd KOS-63-infected mice. This has been in concordance with significant decreased corneal sensation at 5 and 7 dpi. Corneal nerve damage following HSV infection has been well demonstrated in clinical and preclinical studies^7,10,15,16,48,49^. Both KOS and McKrae HSV-1 strains have previously shown to result in nerve degeneration in BALB/c and C57BL/6 mice, respectively^7,10,16,45^. We show that as soon as 1 dpi, Hd HSV-1 KOS-63 and Hd McKrae reduce the central corneal nerve density by 42.34% and 34.10%, as compared to sham-infected C57BL/6 mice, respectively. Previously, our group has shown similar results with McKrae infected mice^10,45^. Further, Chucair-Elliott et al., using the same mouse model, demonstrated a significant decrease in corneal nerves at 6, 8, and 14 dpi with 10^3^ PFU/cornea HSV-1 McKrae infection, as compared to uninfected corneas^7^. Moreover, Yun et al. demonstrated dramatic reduction of corneal nerve fibers in BALB/c mice at 28 dpi with the HSV-1 KOS strain^16^, albeit without quantification of nerve density. Furthermore, quantification methods for nerve density have been qualitative in the latter two studies and are thus not comparable to the quantitative method used herein. The exact cause of denervation has not been elucidated yet, although direct nerve damage by the virus and viral-induced inflammation could be leading causes^10,16^. Based on the current study, higher corneal nerve loss (at 3 dpi) in the Hd-infected group compared to the Ld group can be explained either by a higher corneal viral titer and/or by a higher number of PMNs and NK cells in the Hd-infected mice compared to the Ld-infected mice. On the other hand, more severe nerve loss in the Hd-infected mice as compared to Ld-infected mice may explain more severe neovascularization and immune infiltration into the cornea and not necessarily direct effect of the virus. We have shown an inverse correlation between corneal nerve density and immune cell infiltration in the cornea in clinical and pre-clinical studies^50^. Tepelus et al. also found similar inverse correlation in patients with dry eye disease^51^. Interestingly, Yun et al.^16^ concluded that corneal nerve damage can be maintained even without significant corneal inflammation after HSV-1 infection. These studies assessed nerve loss from different perspectives and still the cause of nerve damage in HSV keratitis remains controversial.

It has been previously reported that the HSV-1 KOS is less virulent than strain 17 and McKrae and has mutated US9 and US8A genes as compared with strain 17^52,53^. Different variants of KOS have been used in laboratories up to date, including KOS-63, KOS-79, and KOS1.1 (KOS (M))^21,54^. In addition, different mutant derivatives of HSV-1 KOS have been used to study the virulence, latency, and reactivation of HSV-1^55–57^. Bowen et al.^54^ demonstrated that despite more than 99.2% similarity among different passages of a same virus (HSV-1 KOS-63), there are small genomic differences. Specifically, the HSV-1 KOS-63 strain that was used for the first time by Dix et al.^21^ is different in 3 genes (proteins), gB (UL27), VP1/2 (UL36), and RR (UL39), as compared to the HSV-1 KOS-63 from Kinchington group (which is used in this study)^54^. UL36 and UL39 proteins were shown previously to influence virulence and replication of HSV-1 virus, respectively^58^. Thus, different sub-strains, mutants, and maybe different passages of HSV-1 KOS used by different laboratories may provide additional explanation for variable clinical scores and the controversy in the reported literature.

Corneal wounding may result in activation of resident leukocytes and infiltration of leukocytes to the cornea^59^. Therefore, in order to eliminate the possibility that scarification creates a confounding factor, we used sham-infected mice as an additional control group, in which scarified corneas were inoculated with PBS. We demonstrate that superficial corneal scarification, which is much less extensive than corneal wounds described by Stepp et al.^59^ do not lead to any significant corneal disease, immune cell infiltration, or corneal nerve damage. Similarly, BenMohamed et al.^12^ and Yuan et al.^60^ did not show any significant eye disease and corneal neovascularization post scarification, respectively. Prior studies have shown that HSV-1 McKrae can infect the murine corneas without scarification, although this has not been shown with the KOS strain^12,29^, thus requiring scarification to study the Ld and Hd KOS-63 strain effects. However, in this study in order to exclude any confounding factor we performed scarification prior to corneal inoculation with HSV-1 KOS-63 or HSV-1 McKrae stains. Given that the primary objective of the study was to compare the Hd and Ld HSV-1 KOS-63 corneal infection, these results were compared to the more virulent McKrae strain that was used as a positive control. We used a modified corneal opacity scoring system, with a more detailed scale than mild, moderate and severe opacity, which has been widely used previously. Furthermore, we used clock hours to report clinical neovascularization. Although this method is sensitive to differentiate subtle changes at different time points post infection but it cannot distinguish the extent of neovascularization. Thus, we chose this scoring system, as our goal was to assess virulence at the acute phase of the disease and to tease out minor changes. Moreover, since we followed infected mice for only 7 days to assess the acute phase of HSV keratitis, we cannot extend our dose-dependent findings to the chronic phase of infection. In addition, the plaque assay that we used for virus titration has the detection limit of 10 PFU.

In conclusion, our results are indicative of ocular virulence of HSV-1 KOS-63 in a dose-dependent fashion when applied on scarified corneas of C57BL/6 mice. Moreover, we showed that HSV-1 KOS-63 dose can affect incidence, viral titers in the cornea and TG, immune cell infiltration to the cornea, and finally corneal nerve damage at different time points pi. Therefore, the choice of a suitable viral strain and viral dose, based on the aims of the study, is of great importance. However, whether the viral load or different immune cell infiltrates (or both) are responsible for more sever clinical manifestations and corneal nerve damage in the Hd versus Ld infected mice is not clear.

## Supplementary information

Supplementary information.

Supplementary Figure 1.

Supplementary Figure 2.

Supplementary Figure 3.

Supplementary Figure 4.
