# Supplementary material for: Herpes simplex virus-1 KOS-63 strain is virulent and causes titer-dependent corneal nerve damage and keratitis
Source: Sci Rep. 2021 Feb 19;11:4267. doi: 10.1038/s41598-021-83412-9 (PMC7895966; doi:10.1038/s41598-021-83412-9)
Supplement: Supplementary file 1 — Supplementary information. [file 41598_2021_83412_MOESM1_ESM.docx]

**Supplementary information**

**Herpes Simplex Virus-1 KOS-63 strain is Virulent and Causes Titer-Dependent Corneal Nerve Damage and Keratitis**

Hamid-Reza Moein,^1,2^ Victor Sendra,^1,2^ Arsia Jamali,^1,2^ Ahmad Kheirkhah,^2^ Deshea L. Harris,^1,2^ Pedram Hamrah^1,2,3†^

^1^Center for Translational Ocular Immunology, Department of Ophthalmology, Tufts Medical Center, Tufts University School of Medicine, Boston, MA; ^2^Schepens Eye Research Institute/Massachusetts Eye and Ear Infirmary, Department of Ophthalmology, Harvard Medical School, Boston, MA; ^3^Cornea Service, New England Eye Center, Tufts Medical Center, Tufts University School of Medicine, Boston, MA

**Supplementary figures:**

**Supplementary Fig. 1**. Comparison of corneal opacity scores in low dose (Ld) and high dose (Hd) HSV-1 KOS-63 and HSV-1 McKrae strains at 1, 3, and 5 days post infection (dpi). **A)** Representative white light pictures from McKrae infected corneas. **B)** Corneal opacity scoring: Corneal opacity was significantly higher at 3dpi (0.25±0.16 vs. 1.25±0.25; *p=0.03) and 5dpi (0.62±0.26 vs. 2.0±0.0; **p=0.001) in Hd as compared to Ld McKrae infected corneas. Bars are showing mean ± SEM. ANOVA: p<0.0001. P values are calculated by one-way ANOVA followed by Tukey’s multiple comparison test.

**Supplementary Fig. 2**. HSV-1 viral titer in corneal and trigeminal ganglia (TG) of HSV-1 McKrae infected mice in comparison with HSV-1 KOS-63 infected mice. **A)** Corneal viral titer is significantly higher in McKrae infected mice as compared to Ld KOS-63 at 3 dpi and Ld and Hd KOS-63 at 7 dpi. **B)** TG viral titer is significantly lower in HSV-1 McKrae infected mice as compared with HSV-1 KOS-63 infected mice at 3 dpi. There was no significant difference at 7 dpi. Bars indicating mean ± SEM. P values are calculated by one-way ANOVA followed by Tukey’s multiple comparison test. ***p<0.0001. NS, non-significant.

**Supplementary Fig. 3**. Number of CD45^+^ inflammatory cells in Hd HSV-1 McKrae infected mice is demonstrated in the central (A) and peripheral (B) cornea in comparison to Ld and Hd HSV-1 KOS-63 infected mice. Bars indicating mean ± SEM. P values are calculated by one-way ANOVA followed by Tukey’s multiple comparison test. ***p<0.0001, *p=0.04. NS, non-significant.

**Supplementary Fig. 4**. Central corneal nerve density in high dose infected HSV-1 McKrae. A) Representative confocal micrographs of whole mount HSV-1 McKrae infected corneas 1 day post infection (dpi), stained with NL637 conjugated beta III tubulin antibody. B) Corneal nerve density (mm/mm^2^) in naïve, sham infected mice, 1dpi KOS-63 and McKrae strains. Corneal nerve density was significantly decreased after high dose KOS-63 and McKrae corneal nerve infection. There was no significant difference between different doses and virus strains. Bars are showing mean ± SEM. Statistical comparisons were calculated by one-way ANOVA followed by Tukey’s multiple comparison test. ¥, demonstrates the statistical significance (p<0.05) between the marked column and naïve cornea. NS, non-significant.
