# Supplementary figures and images for: Herpes simplex virus-1 KOS-63 strain is virulent and causes titer-dependent corneal nerve damage and keratitis
Source: Sci Rep. 2021 Feb 19;11:4267. doi: 10.1038/s41598-021-83412-9 (PMC7895966; doi:10.1038/s41598-021-83412-9)

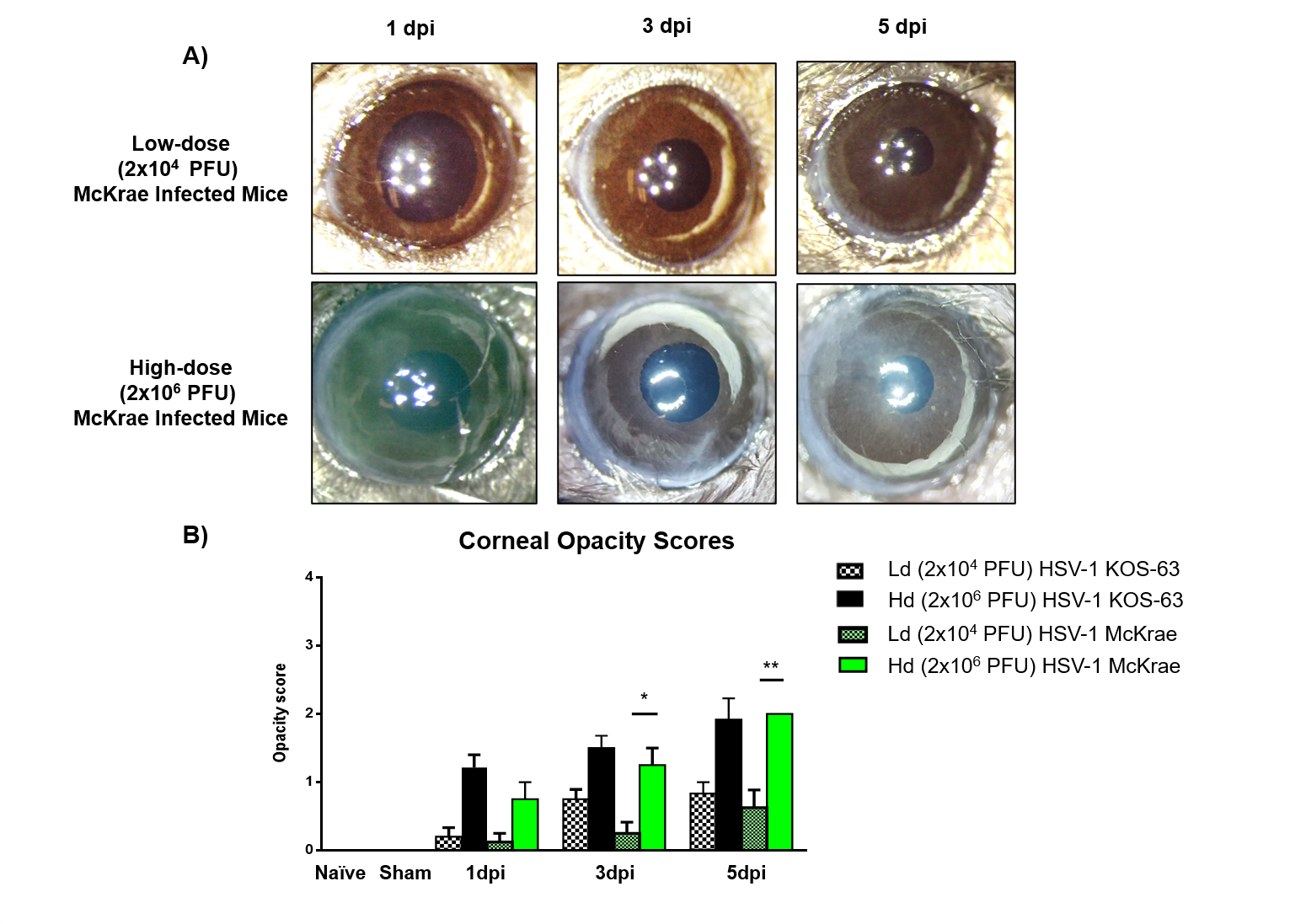

Supplement: Supplementary file 2 — Supplementary Figure 1. [file 41598_2021_83412_MOESM2_ESM.tif]

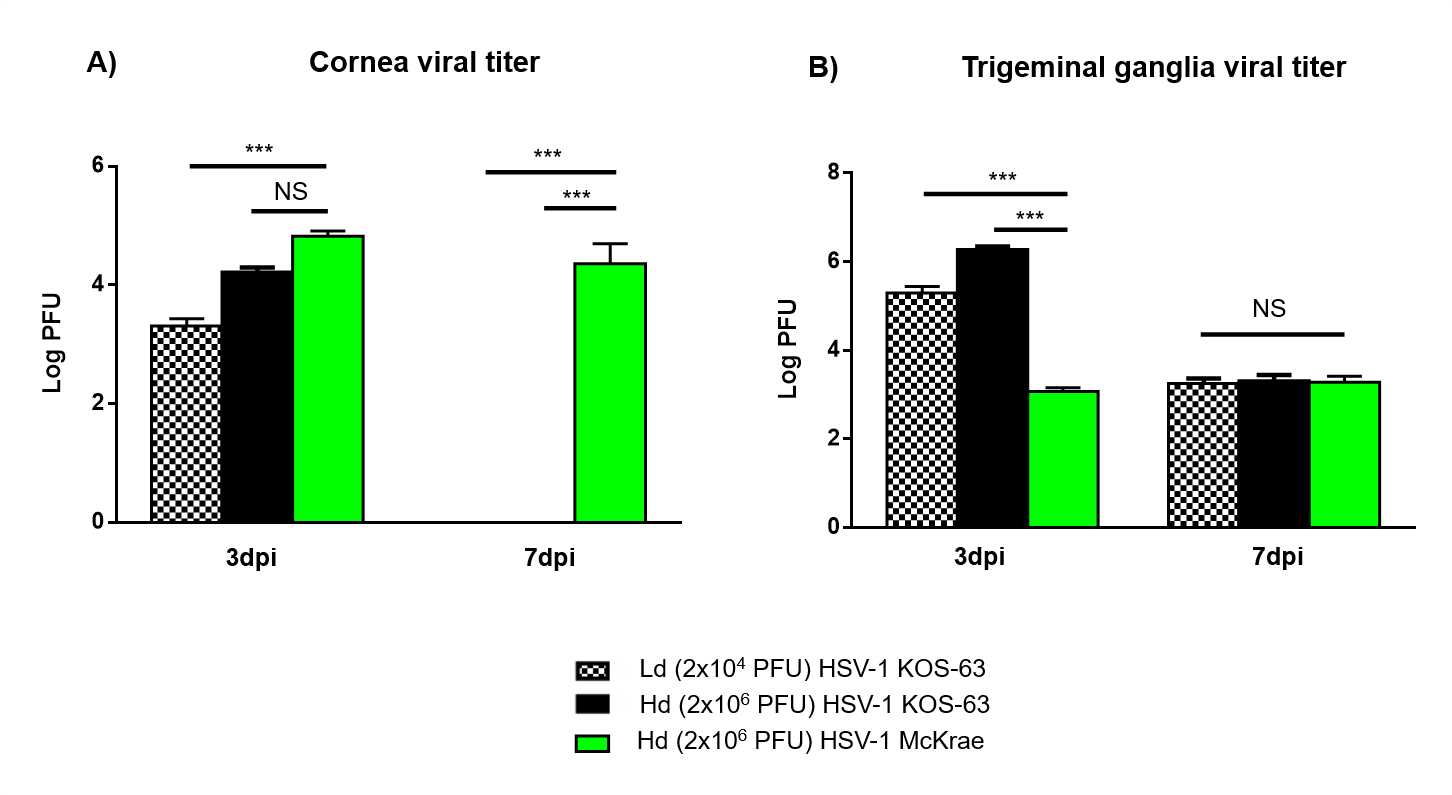

Supplement: Supplementary file 3 — Supplementary Figure 2. [file 41598_2021_83412_MOESM3_ESM.tif]

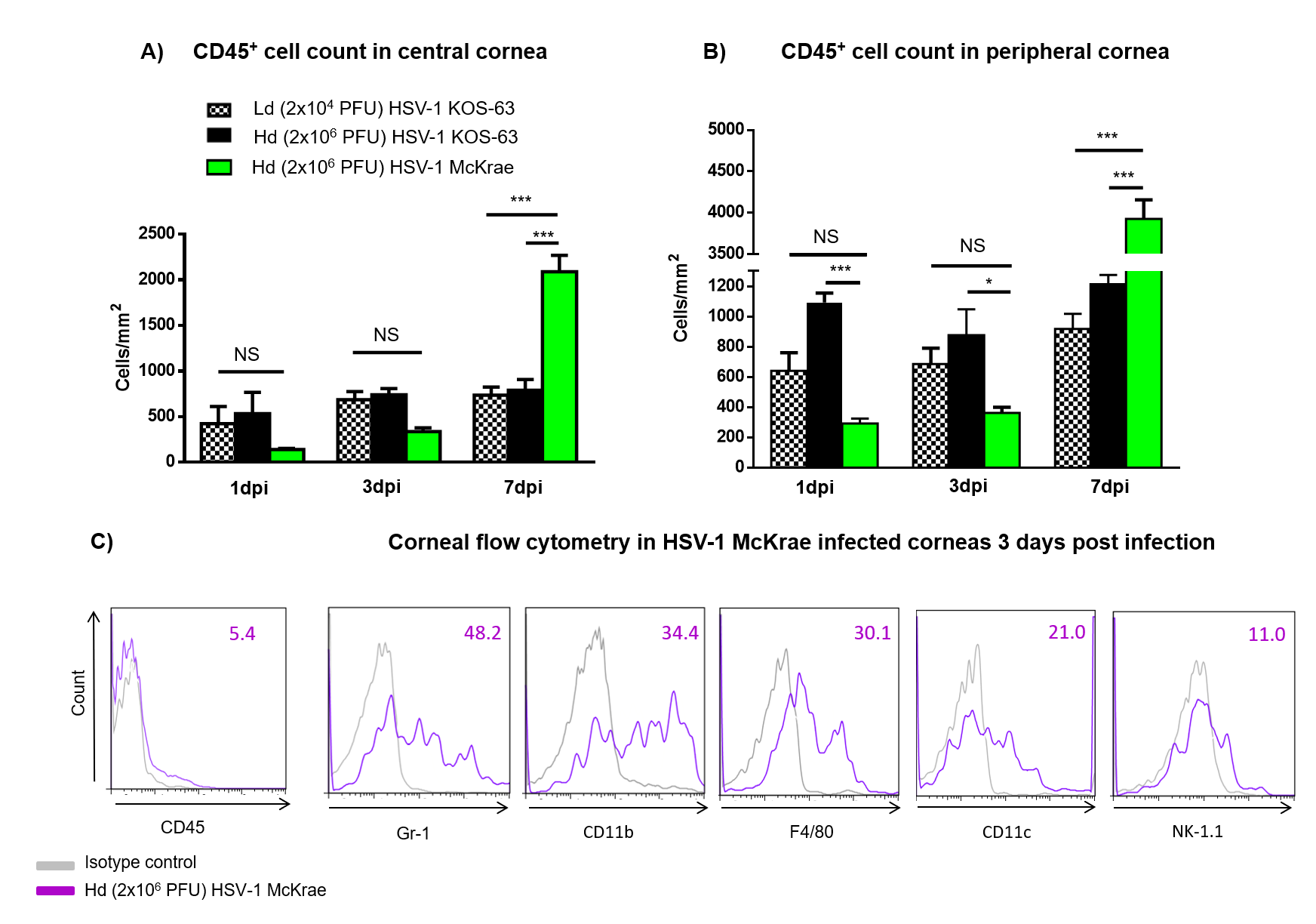

Supplement: Supplementary file 4 — Supplementary Figure 3. [file 41598_2021_83412_MOESM4_ESM.tif]

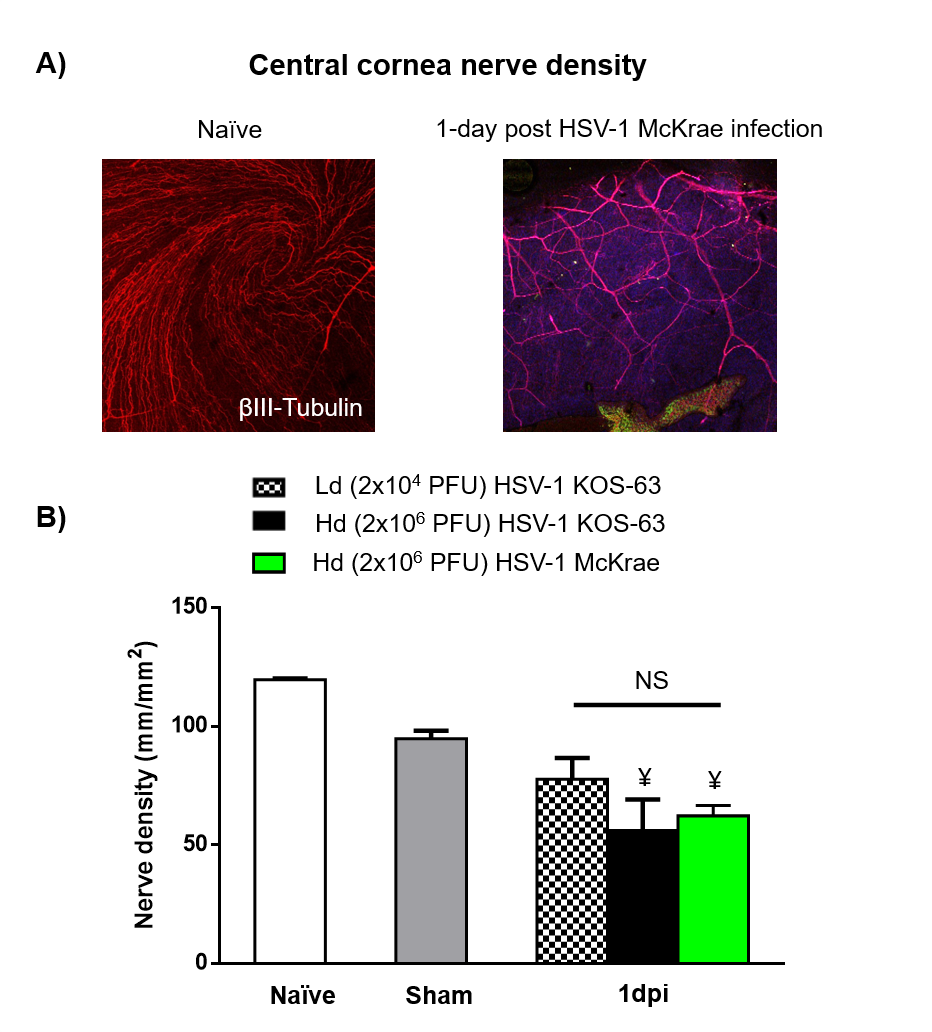

Supplement: Supplementary file 5 — Supplementary Figure 4. [file 41598_2021_83412_MOESM5_ESM.tif]
